# Risk Factors Associated With Transition From Acute to Chronic Low Back Pain in US Patients Seeking Primary Care

**DOI:** 10.1001/jamanetworkopen.2020.37371

**Published:** 2021-02-16

**Authors:** Joel M. Stevans, Anthony Delitto, Samannaaz S. Khoja, Charity G. Patterson, Clair N. Smith, Michael J. Schneider, Janet K. Freburger, Carol M. Greco, Jennifer A. Freel, Gwendolyn A. Sowa, Ajay D. Wasan, Gerard P. Brennan, Stephen J. Hunter, Kate I. Minick, Stephen T. Wegener, Patti L. Ephraim, Michael Friedman, Jason M. Beneciuk, Steven Z. George, Robert B. Saper

**Affiliations:** 1 School of Health and Rehabilitation Sciences, University of Pittsburgh, Pittsburgh, Pennsylvania; 2 Department of Psychiatry, University of Pittsburgh, Pittsburgh, Pennsylvania; 3 Physician Network and Quality, St Clair Hospital, Pittsburgh, Pennsylvania; 4 Department of Physical Medicine and Rehabilitation, University of Pittsburgh, Pittsburgh, Pennsylvania; 5 Department of Anesthesiology and Perioperative Medicine, University of Pittsburgh School of Medicine, Pittsburgh, Pennsylvania; 6 Intermountain Healthcare Rehabilitation Services, Murray, Utah; 7 Johns Hopkins University School of Medicine, Baltimore, Maryland; 8 Department of Epidemiology, Johns Hopkins Bloomberg School of Public Health, Baltimore, Maryland; 9 Johns Hopkins Hospital, Baltimore, Maryland; 10 Department of Physical Therapy, University of Florida College of Public Health and Health Professions, Gainesville; 11 Duke Clinical Research Institute, Department of Orthopedic Surgery, Duke University, Durham, North Carolina; 12 Department of Family Medicine, Boston Medical Center, Boston, Massachusetts

## Abstract

**Importance:**

Acute low back pain (LBP) is highly prevalent, with a presumed favorable prognosis; however, once chronic, LBP becomes a disabling and expensive condition. Acute to chronic LBP transition rates vary widely owing to absence of standardized operational definitions, and it is unknown whether a standardized prognostic tool (ie, Subgroups for Targeted Treatment Back tool [SBT]) can estimate this transition or whether early non–guideline concordant treatment is associated with the transition to chronic LBP.

**Objective:**

To assess the associations between the transition from acute to chronic LBP with SBT risk strata; demographic, clinical, and practice characteristics; and guideline nonconcordant processes of care.

**Design, Setting, and Participants:**

This inception cohort study was conducted alongside a multisite, pragmatic cluster randomized trial. Adult patients with acute LBP stratified by SBT risk were enrolled in 77 primary care practices in 4 regions across the United States between May 2016 and June 2018 and followed up for 6 months, with final follow-up completed by March 2019. Data analysis was conducted from January to March 2020.

**Exposures:**

SBT risk strata and early LBP guideline nonconcordant processes of care (eg, receipt of opioids, imaging, and subspecialty referral).

**Main Outcomes and Measures:**

Transition from acute to chronic LBP at 6 months using the National Institutes of Health Task Force on Research Standards consensus definition of chronic LBP. Patient demographic characteristics, clinical factors, and LBP process of care were obtained via electronic medical records.

**Results:**

Overall, 5233 patients with acute LBP (3029 [58%] women; 4353 [83%] White individuals; mean [SD] age 50.6 [16.9] years; 1788 [34%] low risk; 2152 [41%] medium risk; and 1293 [25%] high risk) were included. Overall transition rate to chronic LBP at six months was 32% (1666 patients). In a multivariable model, SBT risk stratum was positively associated with transition to chronic LBP (eg, high-risk vs low-risk groups: adjusted odds ratio [aOR], 2.45; 95% CI, 2.00-2.98; *P* < .001). Patient and clinical characteristics associated with transition to chronic LBP included obesity (aOR, 1.52; 95% CI, 1.28-1.80; *P* < .001); smoking (aOR, 1.56; 95% CI, 1.29-1.89; *P* < .001); severe and very severe baseline disability (aOR, 1.82; 95% CI, 1.48-2.24; *P* < .001 and aOR, 2.08; 95% CI, 1.60-2.68; *P* < .001, respectively) and diagnosed depression/anxiety (aOR, 1.66; 95% CI, 1.28-2.15; *P* < .001). After controlling for all other variables, patients exposed to 1, 2, or 3 nonconcordant processes of care within the first 21 days were 1.39 (95% CI, 1.21-2.32), 1.88 (95% CI, 1.53-2.32), and 2.16 (95% CI, 1.10-4.25) times more likely to develop chronic LBP compared with those with no exposure (*P* < .001).

**Conclusions and Relevance:**

In this cohort study, the transition rate to chronic LBP was substantial and increased correspondingly with SBT stratum and early exposure to guideline nonconcordant care.

## Key Points

**Question:**

Is the transition from acute to chronic low back pain (LBP) associated with risk strata, defined by a standardized prognostic tool, and/or with early exposure to guideline nonconcordant care?

**Findings:**

In this cohort study of 5233 patients with acute LBP from 77 primary care practices, nearly half the patients were exposed to at least 1 guideline nonconcordant recommendation within the first 21 days after the index visit. Patients were significantly more likely to transition to chronic LBP as their risk on the prognostic tool increased and as they were exposed to more nonconcordant recommendations.

**Meaning:**

In this study, the transition rate to chronic LBP was substantial and increased correspondingly with risk strata and early exposure to guideline nonconcordant care.

## Teaser

This cohort study assesses the associations between the transition from acute to chronic lower back pain with risk strata from a standardized prognostic tool; demographic, clinical, and physician characteristics; and guideline nonconcordant processes of care.

## Introduction

Low back pain (LBP) is the leading cause of disability in the United States, annually accounting for 4.3 million years lived with disability, nearly twice the burden of any other health condition.^1^ Overall, 13% of adults have chronic LBP, with one-third experiencing moderate- to high-impact chronic pain.^2,3^ In the United States, treatment for LBP and related spine disorders now represents the most expensive medical problem, with most costs accrued in ambulatory care settings, including primary care.^4,5^ Chronic LBP contributes most to long-term disability, morbidity, health care, and societal costs, while, acute LBP is given less attention because patients are generally considered to have a favorable prognosis.^6,7^

Recent evidence has questioned the prevailing belief that acute LBP resolves within 3 months.^8,9^ A systematic review indicated that 2% to 48% (median, 26%) of patients with acute LBP in primary care settings transition to chronic LBP.^10^ Wide variability can be attributed to heterogeneous populations and varying operational definitions of acute and chronic LBP. Among a cohort of 605 primary care patients with acute LBP, 9% to 35% were found to have chronic LBP at 6 months depending on chronic LBP operational definitions.^8^ Lacking a standardized definition, research investigating determinants and interventions associated with the transition from acute to chronic LBP is hampered, a main factor that led to convening a National Institute of Health (NIH) Task Force Pain Consortium to develop a standardized definition and research standards for chronic LBP.^11^

The NIH Task Force also recommended further study of prognostic instruments, such as the Subgroups for Targeted Treatment (STarT) Back tool (SBT).^11^ The SBT is a 9-item instrument designed to identify patients with LBP at risk of persistent functional limitations but has not been investigated to assess the transition to chronic LBP. It is also used to guide treatment decisions, whereby minimal care is provided for low-risk patients and more intensive treatment is recommended as risk increases.^12,13^ The SBT is reliable and valid for predicting poor functional outcomes; however, the SBT has not been investigated as a prognostic tool for the acute to chronic LBP transition.^14^

Clinical guidelines consistently recommend reassurance (eg, most episodes of acute LBP resolve quickly and have a very low likelihood of serious underlying pathology) and advice to maintain activity as tolerated.^15,16,17^ Recently, nonpharmacologic interventions, such as heat, massage, acupuncture, or spinal manipulation, are recommended as first-line treatment options, while initial use of diagnostic imaging, specialty consultation, and prescription of opioid medications in the absence of red flags (eg, fever, fracture, malignant neoplasms) are not recommended.^17^ Nonconcordant care can lead to direct and indirect harm, given that it has been linked with medicalization and unnecessary health care utilization.^18,19,20^ Accumulating evidence indicates that guideline-concordant care has not been successfully implemented in primary care; however, the association of nonconcordant care with the transition to chronic LBP remains unclear.^21,22^

To obtain estimates of the transition from acute to chronic LBP, assess the SBT prognostic capabilities, and identify pragmatic factors associated with poor outcomes, we conducted a large multisite inception cohort study. We prospectively enrolled patients with acute LBP who were seen in primary care, administered the SBT at baseline, and assessed for chronic LBP at 6 months using the NIH Task Force definition. The objectives for this study were to assess the association between risk of acute to chronic LBP transition with (1) baseline SBT risk strata; (2) patient demographic and clinical characteristics and practice characteristics; and (3) guideline nonconcordant processes of care.

## Methods

### Study Design and Setting

**Figure 1.**
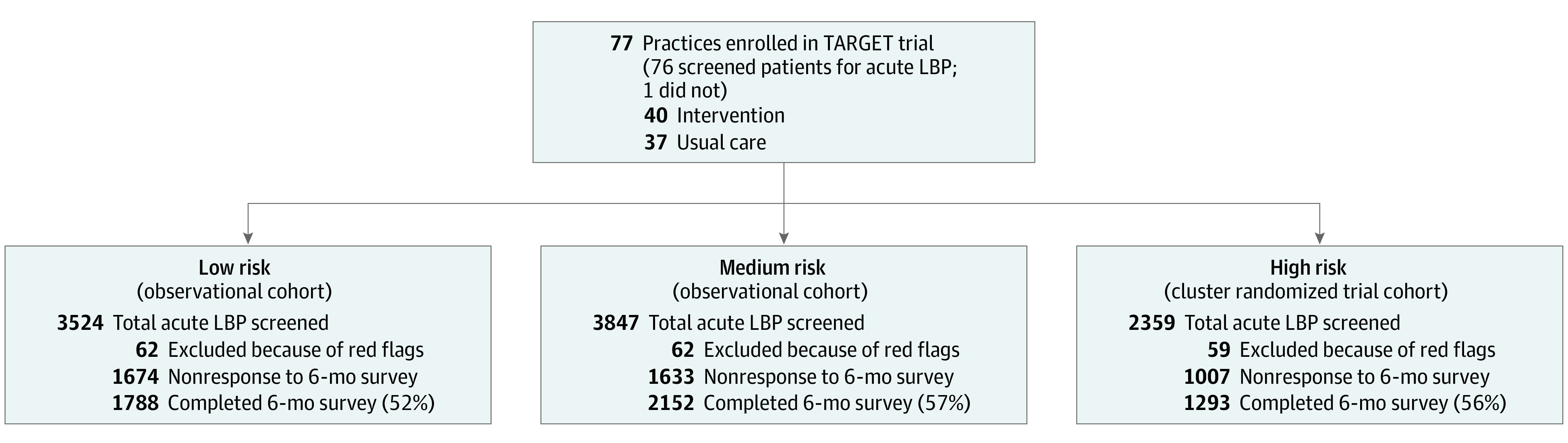
Flow Diagram Depicting Patient Screening for Acute Low Back Pain (LBP) and 6-Month Survey in the Targeted Interventions to Prevent Chronic Low Back Pain in High-Risk Patients (TARGET) Trial Patients who were identified at baseline as having low and medium risk were only included in the observational component of the TARGET study. Patients screened as high risk were included in the observational and the cluster randomized trial component of the TARGET study. In the cluster randomized trial, patients received either usual care or the intervention (usual care with psychologically informed physical therapy) depending on the clinic where they presented.

A detailed study protocol has been published,^23^ and reporting of this manuscript follows the Strengthening the Reporting of Observational Studies in Epidemiology (STROBE) reporting guideline for cohort studies. Briefly, we conducted an inception cohort study alongside the multisite, pragmatic (ie, tested in real-world settings), cluster randomized Targeted Interventions to Prevent Chronic Low Back Pain in High-Risk Patients (TARGET) clinical trial (NCT02647658). Patients presenting to primary care clinics with acute LBP were stratified by risk (ie, low, medium, and high) for developing chronic LBP using the SBT.^24^ High-risk patients were enrolled in the RCT and cohort studies. Medium-risk and low-risk patients were only enrolled in the cohort study. All patients were assessed for the presence or absence of chronic LBP at baseline and 6 months (Figure 1). Patients were enrolled between May 2016 and June 2018 in 77 primary care practices in 4 US health systems (Pittsburgh, Pennsylvania; Boston, Massachusetts; Salt Lake City, Utah; and Baltimore, Maryland), and follow-up was completed by March 2019.

The study was overseen by 4 institutional review boards.^23^ Processes conducted within the primary care clinics were viewed as quality improvement and the 6-month survey as research requiring verbal or written informed consent. Sites had varying approaches to obtaining consent for the 6-month survey. In Boston, Salt Lake City, and Pittsburgh, consent was obtained in conjunction with the 6-month survey, while in Baltimore consent was obtained at baseline.

### Data Collection

All study data except the 6-month surveys were sourced from existing data fields in the electronic medical records (EMRs). EMR data were collected using standardized data extraction and secure file transfer protocols facilitated via honest brokers.

### Cohort Identification

**Figure 2.**
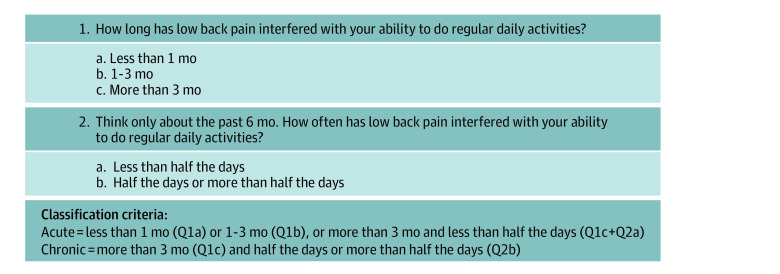
Questionnaire to Classify Acute vs Chronic Low Back Pain This questionnaire has been adapted from the National Institutes of Health Task Force on Research Standards for Chronic Low Back Pain definition. Q indicates question.

Patients were eligible if they were adults (aged ≥18 years) and presented with a primary concern of acute, bothersome axial LBP or LBP with associated leg pain. To determine the nature of the concern, a 2-item acute/chronic LBP screening questionnaire was created by adapting the NIH Task Force on Research Standards chronic LBP definition. Patients were considered to have chronic LBP if they reported (1) the presence of pain for more than 3 months and (2) experienced pain at least half the days in the past 6 months. Those not meeting this definition were classified as having acute LBP (Figure 2). The date the questionnaire was administered was considered the index (ie, baseline) visit. Data were collected via study tablets or verbally by clinical or administrative personnel and uploaded into the EMR. The LBP concern was verified retrospectively using the *International Classification of Diseases, Ninth Revision *(*ICD-9*) or *ICD-10 Clinical Modification* (*ICD-10-CM*) diagnosis codes from the EMR, and patients were excluded if any diagnoses on the index visit indicated a potential red flag for a serious underlying reason for LBP (eg, fracture, cancer).

### Risk Stratification

Risk for developing chronic LBP was determined at the index visit using the 9-item version of the SBT.^24^ The total score ranges from 0 to 9 and includes a psychological subscale score ranging from 0 to 5. Patients were stratified as low-risk (total score ≤3), medium-risk (total score ≥4 and subscale score ≤3), or high risk (total score ≥4 and subscale score ≥4).^24^ The SBT was administered in primary care clinics using the same process as the acute/chronic LBP questionnaire (eFigure 1 in the Supplement).

### Outcome

Chronic LBP status at 6 months was ascertained by the acute/chronic LBP questionnaire. The survey was collected electronically, by mail, or by telephone by research personnel.

### Demographic, Clinical, and Practice Covariates

Patient characteristics included demographic factors (ie, age, sex, race, ethnicity, and health insurance) and clinical characteristics (ie, body mass index, smoking status, LBP diagnosis, psychological comorbidities, self-reported LBP disability). Insurance was collapsed to 4 categories (commercial, Medicare, Medicaid, and other [workers’ compensation, self-pay, missing]). LBP diagnoses and psychological comorbidities were identified via *ICD-9* or *ICD-10-CM* diagnostic codes present at the index visit. LBP disability was assessed via the Oswestry Disability Index (ODI), which was administered at the index visit with the SBT and acute/chronic LBP questionnaire.^25^ The ODI scores range from 0 to 100 and were categorized using the following disability definitions: minimal (0-20), moderate (21-40), severe (41-60), very severe (≥61).^25^ Practice characteristics included geographic location and the national area deprivation index (ADI). The ADI ranks the Census block or neighborhood in terms of socioeconomic disadvantage. We used the validated Neighborhood Atlas Tool to estimate national level ADI scores for each clinic location.^26^

### Nonconcordant Processes of Care

LBP-related processes of care provided by primary care clinicians within 21 days of a patient’s index visit were extracted from the EMR. We used international LBP guidelines and codified these processes of care into 3 categories: pharmacologic therapies, diagnostic imaging, and medical subspecialty referral.^17^ We further categorized each process of care as concordant or nonconcordant with these guidelines. Nonconcordant pharmacotherapy was determined using the algorithm provided in eFigure 2 in the Supplement.^17^ Briefly, any prescriptions that included opioids were considered nonconcordant. Additionally, prescriptions that included benzodiazepines and/or systemic corticosteroids alone without the presence of nonsteroidal anti-inflammatory drugs or short-term skeletal muscle relaxants were considered nonconcordant. Nonconcordant diagnostic imaging consisted of an order for lumbar radiograph or computed tomography/magnetic resonance imaging (CT/MRI) scan. Nonconcordant medical subspecialty referral included referrals to nonsurgical or surgical specialties (eg, physiatrists, orthopedists, neurologists, neurosurgeons, or pain specialists). To improve interpretation and analysis, we created a composite variable that represented the total count of categories with nonconcordant processes of care. The minimum composite score was 0 (ie, patient received no nonconcordant processes of care), while the maximum composite score was 3 (ie, patients received nonconcordant processes of care in all 3 categories).

### Sample Size

Sample size estimates for the high-risk cohort are reported elsewhere.^20^ Estimates for low-risk and medium-risk patients were derived from the proportion of patients expected to screen into these 2 strata in the parent trial’s primary care clinics.^23^ We assumed a mean of 115 patients would screen as low to medium risk per clinic during enrollment of the trial, with 20% transitioning to chronic LBP, an intracluster correlation of 0.01, and a 60% response rate at 6 months. This provided us at minimum a 79% power to detect 30% relative difference in transition to chronic LBP between patient subgroups (eg, SBT stratum) that could be as small as a 1:4 ratio (ie, the subgroup makes up 20% of the sample compared with the remaining 2 subgroups, which make up 80% of the sample).

### Statistical Analysis

We compared baseline demographic, clinical, practice, and process of care characteristics between patients with and without 6-month follow-up surveys using χ^2^ tests. In these comparisons, we adjusted for clustering at the clinic level using Taylor series linearization for variance estimation. We described the rate of transition to chronic LBP at 6 months for each independent variable across the SBT risk stratum and for all strata combined. Univariate and multivariable associations between the independent variables and transition to chronic LBP were assessed using a generalized linear mixed model with a logit link controlling for the cluster design with a random clinic effect. All variables entered into the multivariable models were categorical and treated as fixed effects with significance of *P* < .15 for further consideration. All multivariable models controlled for site as a fixed effect. SBT risk strata and the composite nonconcordant process of care score remained in the model, and a backward stepwise elimination with significance set at *P* < .15 was used to arrive at a final multivariable model. Potential selection bias due to the large proportion of patients with missing 6-month data was addressed by applying stabilized inverse probability weights to the multivariable model.^27^ Data analysis was conducted with SAS version 9.4 (SAS Institute). Statistical significance was set at *P* < .05, and all tests were 2-tailed.

## Results

The final cohort of 5233 patients with acute LBP and 6-month surveys (Figure 1) were mostly women (3029 [58%]) and White individuals (4353 [83%]) with overweight (1599 [31%]) or obesity (2308 [44%]). Most patients were diagnosed with axial LBP (3883 [74%]), and 292 (6%) had an anxiety or depression diagnosis. Risk stratification in the final cohort showed 1788 (34%) were low risk; 2152 (41%), medium risk; and 1293 (25%), high risk for developing chronic LBP. Across risk levels, 1544 patients (30%) received prescriptions for nonrecommended medications (999 [65%] received opioids); 1245 (24%) received an radiography or CT/MRI order; and 333 (6%) were referred to a medical specialist (207 [62%] surgeons) within 21 days of the index visit (Table 1). The survey nonresponse rate was 45% (4314 of 9547). Nonresponders were less likely to be White individuals, to have overweight or obesity, to not currently smoke, to be prescribed nonconcordant pharmacologic therapies, or to receive care from the Intermountain Healthcare or University of Pittsburgh Medical Center systems (Table 1).

**Table 1.** Descriptive Summary of the Inception Cohort

Characteristic	No. (%)			*P* value
	Overall sample (N = 9547)	Missing 6-mo follow-up (n = 4314)	Completed 6-mo follow-up (n = 5233)	
**Patient characteristics**				
Age, y				
18-40	2986 (31)	1282 (30)	1704 (33)	.01
41-60	3697 (39)	1717 (40)	1980 (38)	
≥61	2859 (30)	1310 (30)	1549 (30)	
Sex				
Women	5502 (58)	2473 (57)	3029 (58)	.63
Men	4040 (42)	1836 (43)	2204 (42)	
Race				
White	7587 (80)	3234 (75)	4353 (83)	<.001
Black or African American	1321 (14)	712 (17)	609 (12)	
Other, declined, or not available^a^	634 (7)	363 (8)	271 (5)	
Ethnicity				
Not Hispanic or Latino	8849 (93)	3958 (92)	4891 (93)	.005
Hispanic or Latino	404 (4)	213 (5)	191 (4)	
Declined or not available	289 (3)	138 (3)	151 (3)	
Health insurance				
Private	4888 (51)	2186 (51)	2702 (52)	.27
Medicaid	869 (9)	377 (9)	492 (9)	
Medicare	1923 (20)	871 (20)	1052 (20)	
Workers’ compensation, self-pay, missing, or other	1862 (20)	875 (20)	987 (19)	
Body mass index^b^				
Normal or underweight, <25	1871 (20)	772 (18)	1099 (21)	<.001
Overweight, 25 to <30	2668 (28)	1069 (25)	1599 (31)	
Obesity, ≥30	3757 (39)	1449 (34)	2308 (44)	
Missing or unknown	1246 (13)	1019 (24)	227 (4)	
Smoking				
Not current	6188 (65)	2436 (57)	3752 (72)	<.001
Current	1125 (12)	530 (12)	595 (11)	
Not available	2229 (23)	1343 (31)	886 (17)	
**Clinical characteristics**				
Presenting diagnosis				
Axial back pain	7111 (75)	3228 (75)	3883 (74)	.43
Back and leg pain	2431 (25)	1081 (25)	1350 (26)	
SBT risk level				
Low	3460 (36)	1672 (39)	1788 (34)	<.001
Medium	3783 (40)	1631 (38)	2152 (41)	
High	2299 (24)	1006 (23)	1293 (25)	
Baseline Oswestry Disability Index score				
Minimal, 0-20	2939 (31)	1410 (33)	1529 (29)	.002
Moderate, 21-40	3364 (35)	1476 (34)	1888 (36)	
Severe, 41-60	2063 (22)	880 (20)	1183 (23)	
Very severe, ≥61	912 (10)	422 (10)	490 (9)	
Missing or unknown	264 (3)	121 (3)	143 (3)	
Psychological comorbidities				
None	8973 (94)	4032 (94)	4941 (94)	.07
Depression/anxiety	569 (6)	277 (6)	292 (6)	
**Practice characteristics**				
Geographic location				
UPMC	4907 (51)	2070 (48)	2837 (54)	<.001
Intermountain Healthcare	2068 (22)	663 (15)	1405 (27)	
Johns Hopkins Medicine	1587 (17)	1119 (26)	468 (9)	
Boston Medical Center	980 (10)	457 (11)	523 (10)	
Area deprivation index quartile				
1, Low deprivation	2761 (29)	1255 (29)	1506 (29)	.003
2	1764 (18)	830 (19)	934 (18)	
3	2382 (25)	1001 (23)	1381 (26)	
4, High deprivation	2635 (28)	1223 (28)	1412 (27)	
**Guideline-recommended processes of care**				
Pharmacologic therapies				
Concordant	6925 (73)	3236 (75)	3689 (70)	<.001
Nonconcordant	2617 (27)	1073 (25)	1544 (30)	
Diagnostic imaging				
Concordant	7305 (77)	3317 (77)	3988 (76)	.37
Nonconcordant	2237 (23)	992 (23)	1245 (24)	
Back pain subspecialty referral				
Concordant	8915 (93)	4015 (93)	4900 (94)	.33
Nonconcordant	627 (7)	294 (7)	333 (6)	
Nonconcordant composite				
0	5152 (54)	2430 (56)	2722 (52)	<.001
1	3387 (35)	1449 (34)	1938 (37)	
2	915 (10)	380 (9)	535 (10)	
3	88 (1)	50 (1)	38 (1)	

Abbreviations: SBT, Subgroups for Targeted Treatment Back tool; UPMC, University of Pittsburgh Medical Center.

^a^ Other included American Indian or Alaska Native, Asian, Hawaiian or Pacific Islander, and multirace.

^b^ Body mass index calculated as weight in kilograms divided by height in meters squared.

The overall unadjusted acute to chronic LBP transition rate at 6 months was 32% (1666 of 5233). The unadjusted rates by low-risk, medium-risk, and high-risk stratum were 19% (333), 33% (703), and 49% (630), respectively. Positive univariate associations were found between chronic LBP at 6 months and exposure to nonconcordant pharmacotherapies (606 of 1544 [39%] vs 1060 of 3689 [29%]; *P* < .001), diagnostic imaging (447 of 1245 [36%] vs 1219 of 3988 [31%]; *P* < .001), and referral to medical subspecialists (176 of 333 [53%] vs 1490 of 4900 [30%]; *P* < .001). As the composite nonconcordant processes of care score increased from 0 to 3 , the rates of transition increased in a stepwise fashion from 27% (724 of 2722) to 53% (20 of 38) (*P* < .001) (Table 2).

**Table 2.** Chronic Low Back Pain Status by Independent Variable for Each Risk Level and All Risk Levels Combined for Those With Completed 6-Month Follow-up

Factor	Low risk (n = 1788)		Medium risk (n = 2152)		High risk (n = 1293)		Combined risk levels (N = 5233 )	
	Total, No.	Chronic at 6 mo, No. (%)	Total, No.	Chronic at 6 mo, No. (%)	Total, No.	Chronic at 6 mo, No. (%)	Total, No.	Chronic at 6 mo, No. (%)
**Patient characteristics**								
Age, y^a^								
18-40	580	104 (18)	681	188 (28)	443	214 (48)	1704	506 (30)
41-60	633	124 (20)	838	284 (34)	509	249 (49)	1980	657 (33)
≥61	575	105 (18)	633	231 (36)	341	167 (49)	1549	503 (32)
Sex^a^^,^^b^								
Women	984	176 (18)	1254	429 (34)	791	421 (53)	3029	1026 (34)
Men	804	157 (20)	898	274 (31)	502	209 (42)	2204	640 (29)
Race^a^^,^^b^^,^^c^								
White	1503	282 (19)	1852	581 (31)	998	456 (46)	4353	1319 (30)
Black or African American	187	35 (19)	205	87 (42)	217	133 (61)	609	255 (42)
Other, declined, or not available	98	16 (16)	95	35 (37)	78	41 (53)	271	92 (34)
Ethnicity^a^								
Not Hispanic or Latino	1672	308 (18)	2035	662 (33)	1184	575 (49)	4891	1545 (32)
Hispanic or Latino	60	12 (20)	60	20 (33)	71	38 (54)	191	70 (37)
Declined or not available	56	13 (23)	57	21 (37)	38	17 (45)	151	51 (34)
Health insurance^a^^,^^b^								
Private insurance	965	168 (17)	1177	324 (28)	560	226 (40)	2702	718 (27)
Medicaid	370	67 (18)	457	183 (40)	225	121 (54)	1052	371 (35)
Medicare	134	45 (34)	187	89 (48)	171	108 (63)	492	242 (49)
Workers’ compensation, self-pay, missing, or other	319	53 (17)	331	107 (32)	337	175 (52)	987	335 (34)
Body mass index^a^^,^^b^^,^^d^								
Normal or underweight, <25	428	62 (14)	425	111 (26)	246	112 (46)	1099	285 (26)
Overweight, 25 to <30	574	104 (18)	661	195 (30)	364	155 (43)	1599	454 (28)
Obesity, ≥30	711	156 (22)	959	364 (38)	638	342 (54)	2308	862 (37)
Missing or unknown	75	11 (15)	107	33 (31)	45	21 (47)	227	65 (29)
Smoker^a^^,^^b^								
Not current	1378	245 (18)	1508	486 (32)	866	401 (46)	3752	1132 (30)
Current	138	42 (30)	229	100 (44)	228	138 (61)	595	280 (47)
Not available	272	46 (17)	415	117 (28)	199	91 (46)	886	254 (29)
**Clinical characteristics**								
Presenting diagnosis^a^^,^^b^								
Axial back pain	1476	259 (18)	1521	463 (30)	886	409 (46)	3883	1131 (29)
Back and leg pain	312	74 (24)	631	240 (38)	407	221 (54)	1350	535 (40)
Baseline Oswestry Disability Index score^a^^,^^b^								
Minimal, 0-20	1110	192 (17)	349	78 (22)	70	23 (33)	1529	293 (19)
Moderate, 21-40	520	103 (20)	979	291 (30)	389	148 (38)	1888	542 (29)
Severe, 41-60	99	26 (26)	574	225 (39)	510	271 (53)	1183	522 (44)
Very severe, ≥61	18	3 (17)	193	81 (42)	279	166 (59)	490	250 (51)
Missing or unknown	41	9 (22)	57	28 (49)	45	22 (49)	143	59 (41)
Psychological comorbidities^b^								
None	1690	307 (18)	2042	656 (32)	1209	577 (48)	4941	1540 (31)
Depression/anxiety	98	26 (27)	110	47 (43)	84	53 (63)	292	126 (43)
**Practice characteristics**								
Geographic location^a^^,^^b^								
UPMC	1047	219 (21)	1126	388 (34)	664	327 (49)	2837	934 (33)
Intermountain Healthcare	424	60 (14)	648	184 (28)	333	135 (41)	1405	379 (27)
Johns Hopkins Medicine	143	18 (13)	213	68 (32)	112	47 (42)	468	133 (28)
Boston Medical Center	174	36 (21)	165	63 (38)	184	121 (66)	523	220 (42)
Area Deprivation Index quartile^a^								
1, Low deprivation	529	98 (19)	595	176 (30)	382	200 (52)	1506	474 (31)
2	339	63 (19)	396	125 (32)	199	87 (44)	934	275 (29)
3	471	81 (17)	582	209 (36)	328	150 (46)	1381	440 (32)
4, High deprivation	449	91 (20)	579	193 (33)	384	193 (50)	1412	477 (34)
**Guideline-recommended processes of care**								
Pharamcologic therapies^a^^,^^b^								
Concordant	1439	249 (17)	1405	415 (30)	845	396 (47)	3689	1060 (29)
Nonconcordant	349	84 (24)	747	288 (39)	448	234 (52)	1544	606 (39)
Diagnostic imaging^a^^,^^b^								
Concordant	1416	254 (18)	1616	500 (31)	956	465 (49)	3988	1219 (31)
Nonconcordant	372	79 (21)	536	203 (38)	337	165 (49)	1245	447 (36)
Back pain subspecialty referral^a^^,^^b^								
Concordant	1713	308 (18)	2027	634 (31)	1160	548 (47)	4900	1490 (30)
Nonconcordant	75	25 (33)	125	69 (55)	133	82 (62)	333	176 (53)
Nonconcordant composite^a^^,^^b^								
0	1106	180 (16)	1018	269 (26)	598	275 (46)	2722	724 (27)
1	572	119 (21)	877	317 (36)	489	239 (49)	1938	675 (35)
2	106	33 (31)	240	108 (45)	189	106 (56)	535	247 (46)
3	4	1 (25)	17	9 (53)	17	10 (59)	38	20 (53)

Abbreviation: UPMC, University of Pittsburgh Medical Center.

^a^ Overall term was significantly associated with Subgroups for Targeted Treatment Back tool risk level at *P* < .05.

^b^ Overall term was significant in a univariate logistic regression model controlling for the clustered study design of chronic low back pain status at 6 months in the combined risk levels at *P* < .05.

^c^ Other included American Indian or Alaska Native, Asian, Hawaiian or Pacific Islander, and multirace.

^d^ Body mass index calculated as weight in kilograms divided by height in meters squared.

### Factors Associated With Transition to Chronic LBP From the Multivariable Model

In the multivariable model, SBT risk strata were positively associated with the development of chronic LBP when controlling for all other variables. Compared with patients in the low-risk category, the adjusted odds ratio (aOR) of developing chronic LBP was 2.45 (95% CI, 2.00-2.98) times higher for those in the high-risk category and 1.59 (95% CI, 1.33-1.89) times higher for those in the medium-risk category (*P* < .001) (Table 3). Furthermore, there was a stepwise linear relationship across each SBT stratum (*P *for trend < .001).

**Table 3.** Results of the Final Multivariable Logistic Regression Models for Transition From Acute to Chronic Low Back Pain for Those With Complete 6-Month Follow-Up and Adjusted by Inverse Probability Weighting

Factor	Complete 6-mo follow-up		Inverse probability weighted^a^	
	aOR (95% CI)	*P* value	aOR (95% CI)	*P* value
**Patient characteristics**				
Race				
White	1 [Reference]	.05	1 [Reference]	.17
Black or African American	1.31 (1.04-1.65)		1.22 (0.98-1.52)	
Other, declined, or not available^b^	1.25 (0.93-1.69)		1.18 (0.89-1.55)	
Health insurance				
Private insurance	1 [Reference]	<.001	1 [Reference]	<.001
Medicaid	1.91 (1.53-2.38)		1.88 (1.50-2.36)	
Medicare	1.43 (1.21-1.69)		1.54 (1.30-1.82)	
Workers’ compensation, self-pay, missing, or other	1.05 (0.87-1.26)		1.08 (0.90-1.29)	
Body mass index^c^				
Normal or underweight, <25	1 [Reference]	<.001	1 [Reference]	<.001
Overweight, 25 to <30	1.12 (0.93-1.35)		1.57 (1.31-1.87)	
Obesity, ≥30	1.52 (1.28-1.80)		1.53 (1.28-1.83)	
Missing or unknown	1.15 (0.82-1.62)		1.24 (0.96-1.60)	
Smoker				
Not current	1 [Reference]	<.001	1 [Reference]	<.001
Current	1.56 (1.29-1.89)		1.63 (1.35-1.97)	
Not available	1.10 (0.90-1.36)		1.15 (0.93-1.41)	
**Clinical characteristics**				
Presenting diagnosis				
Axial back pain	1 [Reference]	.04	1 [Reference]	.03
Back and leg pain	1.16 (1.00-1.35)		1.17 (1.01-1.36)	
SBT risk level				
Low	1 [Reference]	<.001	1 [Reference]	<.001
Medium	1.59 (1.33-1.89)		1.63 (1.37-1.94)	
High	2.45 (2.00-2.98)		2.52 (2.06-3.07)	
Baseline Oswestry Disability Index score				
Minimal, 0-20	1 [Reference]	<.001	1 [Reference]	<.001
Moderate, 21-40	1.16 (0.97-1.39)		1.14 (0.95-1.37)	
Severe, 41-60	1.82 (1.48-2.24)		1.85 (1.50-2.28)	
Very severe, ≥61	2.08 (1.60-2.68)		1.83 (1.49-2.26)	
Missing or unknown	1.90 (1.29-2.80)		1.66 (1.12-2.46)	
Psychological comorbidities				
None	1 [Reference]	<.001	1 [Reference]	<.001
Depression/anxiety	1.66 (1.28-2.15)		1.73 (1.32-2.23)	
**Practice characteristics**				
Geographic location				
UPMC	1 [Reference]	.08	1 [Reference]	.01
Intermountain Healthcare	0.80 (0.64-0.99)		0.80 (0.65-1.00)	
Johns Hopkins Medicine	0.86 (0.64-1.16)		0.88 (0.67-1.16)	
Boston Medical Center	1.19 (0.89-1.59)		1.35 (1.03-1.77)	
**Guideline recommended processes of care**				
Nonconcordant composite				
0	1 [Reference]	<.001	1 [Reference]	<.001
1	1.39 (1.21-2.32)		1.41 (1.22-1.63)	
2	1.88 (1.53-2.32)		1.90 (1.53-2.36)	
3	2.16 (1.10-4.25)		2.03 (1.01-4.08)	

Abbreviations: aOR, adjusted odds ratio; SBT, Subgroups for Targeted Treatment Back tool; UPMC, University of Pittsburgh Medical Center.

^a^ The median (interquartile range) of the inverse probability weights equaled 0.94 (0.49-6.15).

^b^ Other included American Indian or Alaska Native, Asian, Hawaiian or Pacific Islander, and multirace.

^c^ Body mass index calculated as weight in kilograms divided by height in meters squared.

Baseline disability was positively associated with transition to chronic LBP. The aOR for developing chronic LBP was 1.16 (95% CI, 0.97-1.39) times higher for moderate disability, 1.82 (95% CI, 1.48-2.24) times higher for severe disability, and 2.08 (95% CI, 1.60-2.68) times higher for very severe disability compared with minimal disability (*P* < .001) (Table 3). Other significant independent factors included health insurance (eg, Medicaid: aOR, 1.91; 95% CI, 1.53-2.38; *P* < .001), body mass index (eg, obesity: aOR, 1.52; 95% CI, 1.28-1.80; *P* < .001), smoking status (aOR, 1.56; 95% CI, 1.29-1.89; *P* < .001), diagnosis at the index visit (back and leg pain: aOR, 1.16; 95% CI, 1.00-1.35; *P* = .04), and psychological comorbidities (aOR, 1.66; 95% CI, 1.28-2.15; *P* < .001).

Exposure to nonconcordant care was associated with increased odds of developing chronic LBP (*P* < .001). The aORs for developing chronic LBP were 1.39 (95% CI, 1.21-2.32), 1.88 (95% CI, 1.53-2.32), and 2.16 (95% CI, 1.10-4.25) times higher for exposure to 1, 2, or 3 nonconcordant processes of care, respectively, compared with 0 nonconcordant processes of care (*P* < .001) (Table 3). Additionally, there was a positive linear association between the number of nonconcordant processes of care and risk of developing chronic LBP (*P* for trend = .04).

### Inverse Probability Weighted Multivariable Model

The multivariable model was reexamined using stabilized inverse probability weighting, and the results closely matched the direction and magnitude of the aORs in the original model, except for minor differences in the width of the confidence intervals (Table 3). The linear associations between the transition to chronic LBP and SBT strata (*P* for trend < .001) and the number of nonconcordant processes of care (*P *for trend = .02) remained significant.

## Discussion

We present the results of a large prospective, multicenter study conducted to determine the proportion of patients who transitioned from acute to chronic LBP in primary care settings across 4 geographically dispersed health systems. Based on the NIH Task Force definition, the overall transition to chronic LBP was 32%. The risk of transition was linearly associated with baseline SBT category and whether early care was nonconcordant with current practice guidelines. Collectively, these results indicate that the transition from acute to chronic LBP is much greater than historically appreciated, the SBT can estimate risk of transition, and lack of guideline adherence may increase transition rates. These results expand SBT’s capabilities to include the transition to chronic LBP using the NIH operational definition and reinforce the importance of LBP guidelines.

Practice guidelines do not consistently recommend the use of risk stratification tools, such as the SBT, for acute LBP, likely due to the prevailing perception that acute LBP has a favorable prognosis.^17^ The SBT was designed to tailor treatments based on risk of persistent functional limitations. In clinical practice, it may be tempting to focus on high-risk groups given the high transition rate to chronic LBP. However, it should be noted that more than 60% of the 1666 patients who developed chronic LBP at 6 months were in the low-risk (333 patients) and medium-risk (703) groups. Even though the rate of transition to chronic LBP is lower in these groups, most patients with acute LBP (>75%) fall into these strata. As a result, uniformly applying a minimalist approach (eg, advice, reassurance) to all patients with acute LBP without considering SBT risk status is unwarranted and may lead to suboptimal care.^17^ Conversely, uniformly administering resource intensive, multimodal interventions across the entire acute LBP population is unwarranted and may result in low-value care.^5,28^ To ensure appropriate treatment intensity and cost-effectiveness, future research should consider both patient phenotype and the prevalence within each SBT stratum to identify effective and scalable interventions.^29,30^

In this cohort, patient demographic and clinical factors associated with the chronic LBP transition included obesity, smoking, insurance coverage, LBP with leg pain, baseline disability, and diagnosed depression/anxiety. The role that these factors play in the transition to chronic LBP cannot be ignored; however, many of these factors are difficult to change or nonmodifiable altogether. Importantly, our findings demonstrate that independent of these factors, exposure to nonconcordant processes of care during the early phase of treatment was associated with developing chronic LBP. Nearly half of patients (48%) received at least 1 discordant process of care within 3 weeks of the index visit. Even after controlling for patient characteristics (eg, obesity) and clinical characteristics (eg, baseline disability), increasing numbers of nonconcordant management approaches increased the likelihood of having chronic LBP at 6 months. These rates of nonconcordant processes of care are similar to those reported in a claims analysis from 2.5 million individuals newly diagnosed with LBP.^5^ The independent association between nonconcordant care and risk of developing chronic LBP highlights the need to identify strategies that facilitate LBP guideline implementation.

Successful management of LBP is a vexing problem, and health systems have been challenged to develop innovative solutions.^29,31^ Once chronic, LBP is particularly problematic to manage; thus, preventing the transition from acute to chronic LBP is important. Primary care physicians worldwide are under enormous pressure to do more with less, which is the basis for the SBT risk stratification approach.^32^ However, LBP guidelines have yet to consistently recommend the use of risk stratification, and implementing this approach in primary care is proving to be difficult.^33,34^ One reason for poor implementation may trace back to physicians’ musculoskeletal training.^35^ Medical educators have recognized for years that training in musculoskeletal medicine is suboptimal for medical students, residents, and general practitioners.^35,36,37^ Placing a greater emphasis on a highly prevalent condition, such as LBP, during training may improve implementation at the individual level.

Other possible reasons for poor implementation include high caseloads and the overwhelming volume of guidelines directed at primary care; typical physicians would need an estimated 18 hours per day to address all guideline recommendations.^38^ It is time to test supportive models of care to assist primary care practitioners in addressing this substantial public health problem. Evidence from other conditions suggests that organizational strategies that incorporate nonphysician health professionals (eg, nurse practitioners or physician assistants) to comanage cases can improve guideline adherence in primary care.^39^ The Primary Spine Practitioner is another model proposed in the United States in which chiropractors and physical therapists serve as the initial or early point of contact for patients with LBP.^40^ Another potentially beneficial organizational strategy is the use of multidisciplinary teams comprised of medical specialists and other health professionals (eg, integrated practice units).^41,42,43^ Future studies need to evaluate whether different models of care in conjunction with risk stratification can improve guideline concordance, patient outcomes, and decrease the total cost of care.

### Limitations

This study has limitations. We used survey methods to collect 6-month outcomes due to the large sample size and pragmatic nature of the study. Our response rate was 55%, which would be considered low by standards of a clinical efficacy trial. However, response rates in the range of 50% to 60% are considered to pose minimal risk of nonresponse bias when using survey methods. In addition, our inverse probability weighted analysis accounted for selection bias due to nonresponse and confirmed our original conclusions.^27,44,45^

In this study, we relied on EMR data to develop the clinical profile of patients with acute LBP. Although widely generalizable, EMR data may not reflect all pertinent clinical findings, conditions, or comorbidities considered or addressed by the physician, nor do they include all potential confounders that may affect the transition to chronic LBP or necessitate deviation from guidelines. For example, nonconcordant care may be provided to patients with more complex acute LBP. We cannot completely rule out residual confounding; however, we controlled for a broad set of factors associated with higher clinical severity (baseline disability and LBP with leg pain) and factors that complicate management (body mass index, smoking, psychological comorbidities, and SBT risk status).^46^

## Conclusions

This large inception cohort study found that the transition from acute to chronic LBP was substantial and the SBT was a robust prognostic tool. Early exposure to guideline nonconcordant care was significantly and independently associated with the transition to chronic LBP after accounting for patient demographic and clinical characteristics, such as obesity, smoking, baseline disability, and psychological comorbidities. These findings suggest that an emphasis should be placed on discovering strategies to successfully implement guideline concordant care in the primary care setting to reduce the development of chronic LBP.

## Notes

Supplement. **eFigure 1.** Start Back Screening Tool **eFigure 2.** Algorithm to Classify Guideline Nonconcordant Care for Pharmacologic Therapies

## References

[1] Global Burden of Disease Collaborative Network Global Burden of Disease Study 2017 (GBD 2017) results. Accessed January 11, 2021. http://ghdx.healthdata.org/gbd-results-tool

[2] Von Korff M, Scher AI, Helmick C, United States National Pain Strategy for population research: concepts, definitions, and pilot data. J Pain. 2016;17(10):1068-1080. doi:10.1016/j.jpain.2016.06.009 27377620

[3] Dahlhamer J, Lucas J, Zelaya C, Prevalence of chronic pain and high-impact chronic pain among adults—United States, 2016. MMWR Morb Mortal Wkly Rep. 2018;67(36):1001-1006. doi:10.15585/mmwr.mm6736a2 30212442 PMC6146950

[4] Dieleman JL, Cao J, Chapin A, US health care spending by payer and health condition, 1996-2016. JAMA. 2020;323(9):863-884. doi:10.1001/jama.2020.0734 32125402 PMC7054840

[5] Kim LH, Vail D, Azad TD, Expenditures and health care utilization among adults with newly diagnosed low back and lower extremity pain. JAMA Netw Open. 2019;2(5):e193676. doi:10.1001/jamanetworkopen.2019.3676 31074820 PMC6512284

[6] Pengel LH, Herbert RD, Maher CG, Refshauge KM Acute low back pain: systematic review of its prognosis. BMJ. 2003;327(7410):323. doi:10.1136/bmj.327.7410.323 12907487 PMC169642

[7] Koes BW, van Tulder MW, Thomas S Diagnosis and treatment of low back pain. BMJ. 2006;332(7555):1430-1434. doi:10.1136/bmj.332.7555.1430 16777886 PMC1479671

[8] Mehling WE, Gopisetty V, Bartmess E, The prognosis of acute low back pain in primary care in the United States: a 2-year prospective cohort study. Spine (Phila Pa 1976). 2012;37(8):678-684. doi:10.1097/BRS.0b013e318230ab20 22504516 PMC3335773

[9] Itz CJ, Geurts JW, van Kleef M, Nelemans P Clinical course of non-specific low back pain: a systematic review of prospective cohort studies set in primary care. Eur J Pain. 2013;17(1):5-15. doi:10.1002/j.1532-2149.2012.00170.x 22641374

[10] Chou R, Shekelle P Will this patient develop persistent disabling low back pain? JAMA. 2010;303(13):1295-1302. doi:10.1001/jama.2010.344 20371789

[11] Deyo RA, Dworkin SF, Amtmann D, Report of the NIH Task Force on research standards for chronic low back pain. J Pain. 2014;15(6):569-585. doi:10.1016/j.jpain.2014.03.005 24787228 PMC4128347

[12] Hill JC, Whitehurst DG, Lewis M, Comparison of stratified primary care management for low back pain with current best practice (STarT Back): a randomised controlled trial. Lancet. 2011;378(9802):1560-1571. doi:10.1016/S0140-6736(11)60937-9 21963002 PMC3208163

[13] Foster NE, Mullis R, Hill JC, ; IMPaCT Back Study team Effect of stratified care for low back pain in family practice (IMPaCT Back): a prospective population-based sequential comparison. Ann Fam Med. 2014;12(2):102-111. doi:10.1370/afm.1625 24615305 PMC3948756

[14] Hill JC, Dunn KM, Main CJ, Hay EM Subgrouping low back pain: a comparison of the STarT Back Tool with the Orebro Musculoskeletal Pain Screening Questionnaire. Eur J Pain. 2010;14(1):83-89. doi:10.1016/j.ejpain.2009.01.003 19223271 PMC2809923

[15] Koes BW, van Tulder MW, Ostelo R, Kim Burton A, Waddell G Clinical guidelines for the management of low back pain in primary care: an international comparison. Spine (Phila Pa 1976). 2001;26(22):2504-2513. doi:10.1097/00007632-200111150-00022 11707719

[16] Koes BW, van Tulder M, Lin CW, Macedo LG, McAuley J, Maher C An updated overview of clinical guidelines for the management of non-specific low back pain in primary care. Eur Spine J. 2010;19(12):2075-2094. doi:10.1007/s00586-010-1502-y 20602122 PMC2997201

[17] Oliveira CB, Maher CG, Pinto RZ, Clinical practice guidelines for the management of non-specific low back pain in primary care: an updated overview. Eur Spine J. 2018;27(11):2791-2803. doi:10.1007/s00586-018-5673-2 29971708

[18] Tucker HR, Scaff K, McCloud T, Harms and benefits of opioids for management of non-surgical acute and chronic low back pain: a systematic review. Br J Sports Med. 2020;54(11):664. doi:10.1136/bjsports-2018-099805 30902816

[19] Lemmers GPG, van Lankveld W, Westert GP, van der Wees PJ, Staal JB Imaging versus no imaging for low back pain: a systematic review, measuring costs, healthcare utilization and absence from work. Eur Spine J. 2019;28(5):937-950. doi:10.1007/s00586-019-05918-1 30796513

[20] Jacobs JC, Jarvik JG, Chou R, Observational study of the downstream consequences of inappropriate MRI of the lumbar spine. J Gen Intern Med. 2020;35(12):3605-3612. doi:10.1007/s11606-020-06181-7 32989711 PMC7728897

[21] Mafi JN, McCarthy EP, Davis RB, Landon BE Worsening trends in the management and treatment of back pain. JAMA Intern Med. 2013;173(17):1573-1581. doi:10.1001/jamainternmed.2013.8992 23896698 PMC4381435

[22] Kamper SJ, Logan G, Copsey B, What is usual care for low back pain? a systematic review of health care provided to patients with low back pain in family practice and emergency departments. Pain. 2020;161(4):694-702. doi:10.1097/j.pain.0000000000001751 31738226

[23] Delitto A, Patterson CG, Stevans JM, Study protocol for targeted interventions to prevent chronic low back pain in high-risk patients: a multi-site pragmatic cluster randomized controlled trial (TARGET Trial). Contemp Clin Trials. 2019;82:66-76. doi:10.1016/j.cct.2019.05.010 31136834

[24] Hill JC, Dunn KM, Lewis M, A primary care back pain screening tool: identifying patient subgroups for initial treatment. Arthritis Rheum. 2008;59(5):632-641. doi:10.1002/art.23563 18438893

[25] Fairbank JC, Pynsent PB The Oswestry Disability Index. Spine (Phila Pa 1976). 2000;25(22):2940-2952. doi:10.1097/00007632-200011150-00017 11074683

[26] Neighborhood Atlas. Accessed January 11, 2021. https://www.neighborhoodatlas.medicine.wisc.edu/mapping

[27] Seaman SR, White IR Review of inverse probability weighting for dealing with missing data. Stat Methods Med Res. 2013;22(3):278-295. doi:10.1177/0962280210395740 21220355

[28] Atlas SJ Management of low back pain: getting from evidence-based recommendations to high-value care. Ann Intern Med. 2017;166(7):533-534. doi:10.7326/M17-0293 28192792

[29] Traeger AC, Buchbinder R, Elshaug AG, Croft PR, Maher CG Care for low back pain: can health systems deliver? Bull World Health Organ. 2019;97(6):423-433. doi:10.2471/BLT.18.226050 31210680 PMC6560373

[30] George SZ, Goertz C, Hastings SN, Fritz JM Transforming low back pain care delivery in the United States. Pain. 2020;161(12):2667-2673. doi:10.1097/j.pain.0000000000001989 32694378 PMC7669560

[31] Buchbinder R, van Tulder M, Öberg B, ; Lancet Low Back Pain Series Working Group Low back pain: a call for action. Lancet. 2018;391(10137):2384-2388. doi:10.1016/S0140-6736(18)30488-4 29573871

[32] Korownyk C, McCormack J, Kolber MR, Garrison S, Allan GM Competing demands and opportunities in primary care. Can Fam Physician. 2017;63(9):664-668. 28904027 PMC5597006

[33] Sowden G, Hill JC, Morso L, Louw Q, Foster NE Advancing practice for back pain through stratified care (STarT Back). Braz J Phys Ther. 2018;22(4):255-264. doi:10.1016/j.bjpt.2018.06.003 29970301 PMC6095099

[34] Cherkin D, Balderson B, Wellman R, Effect of low back pain risk-stratification strategy on patient outcomes and care processes: the MATCH randomized trial in primary care. J Gen Intern Med. 2018;33(8):1324-1336. doi:10.1007/s11606-018-4468-9 29790073 PMC6082187

[35] Day CS, Yeh AC, Franko O, Ramirez M, Krupat E Musculoskeletal medicine: an assessment of the attitudes and knowledge of medical students at Harvard Medical School. Acad Med. 2007;82(5):452-457. doi:10.1097/ACM.0b013e31803ea860 17457065

[36] Finestone AS, Raveh A, Mirovsky Y, Lahad A, Milgrom C Orthopaedists’ and family practitioners’ knowledge of simple low back pain management. Spine (Phila Pa 1976). 2009;34(15):1600-1603. doi:10.1097/BRS.0b013e3181a96622 19564770

[37] Matzkin E, Smith EL, Freccero D, Richardson AB Adequacy of education in musculoskeletal medicine. J Bone Joint Surg Am. 2005;87(2):310-314. doi:10.2106/00004623-200502000-00011 15687152

[38] Allan GM, McCormack JP, Korownyk C, Lindblad AJ, Garrison S, Kolber MR The future of guidelines: primary care focused, patient oriented, evidence based and simplified. Maturitas. 2017;95:61-62. doi:10.1016/j.maturitas.2016.08.015 27612638

[39] Lau R, Stevenson F, Ong BN, Achieving change in primary care—effectiveness of strategies for improving implementation of complex interventions: systematic review of reviews. BMJ Open. 2015;5(12):e009993. doi:10.1136/bmjopen-2015-009993 26700290 PMC4691771

[40] Murphy DR, Justice BD, Paskowski IC, Perle SM, Schneider MJ The establishment of a primary spine care practitioner and its benefits to health care reform in the United States. Chiropr Man Therap. 2011;19(1):17. doi:10.1186/2045-709X-19-17 21777444 PMC3154851

[41] Fox J, Haig AJ, Todey B, Challa S The effect of required physiatrist consultation on surgery rates for back pain. Spine (Phila Pa 1976). 2013;38(3):E178-E184. doi:10.1097/BRS.0b013e31827bf40c 23138405

[42] Standaert CJ, Li JW, Glassman SJ, Costs associated with the treatment of low back disorders: a comparison of surgeons and physiatrists. PM R. 2020;12(6):551-562. doi:10.1002/pmrj.12266 31628773

[43] van Harten WH Turning teams and pathways into integrated practice units: appearance characteristics and added value. Int J Care Coord. 2018;21(4):113-116. doi:10.1177/2053434518816529 30595841 PMC6297896

[44] Draugalis JR, Plaza CM Best practices for survey research reports revisited: implications of target population, probability sampling, and response rate. Am J Pharm Educ. 2009;73(8):142. doi:10.5688/aj7308142 20221335 PMC2828303

[45] Morton SM, Bandara DK, Robinson EM, Carr PE In the 21st Century, what is an acceptable response rate? Aust N Z J Public Health. 2012;36(2):106-108. doi:10.1111/j.1753-6405.2012.00854.x 22487341

[46] George SZ, Lentz TA, Beneciuk JM, Bhavsar NA, Mundt JM, Boissoneault J Framework for improving outcome prediction for acute to chronic low back pain transitions. Pain Rep. 2020;5(2):e809. doi:10.1097/PR9.0000000000000809 32440606 PMC7209816

