# Supplementary material for: Risk Factors Associated With Transition From Acute to Chronic Low Back Pain in US Patients Seeking Primary Care
Source: JAMA Netw Open. 2021 Feb 16;4(2):e2037371. doi: 10.1001/jamanetworkopen.2020.37371 (PMC7887659; doi:10.1001/jamanetworkopen.2020.37371)
Supplement: Supplement. — eFigure 1. Start Back Screening Tool eFigure 2. Algorithm to Classify Guideline Nonconcordant Care for Pharmacologic Therapies [file jamanetwopen-e2037371-s001.pdf]

Supplemental Online Content

Stevans JM, Delitto A, Khoja SS, et al. Risk Factors Associated With Transition From Acute to Chronic Low Back Pain in US Patients Seeking Primary Care. *JAMA Netw Open*. 2021;4(2):e2037371. doi:10.1001/jamanetworkopen.2020.37371

eFigure 1. Start Back Screening Tool

eFigure 2. Algorithm to Classify Guideline Nonconcordant Care for Pharmacologic Therapies

This supplemental material has been provided by the authors to give readers additional information about their work.

© 2021 Stevans JM et al. *JAMA Network Open.*

eFigure 1. Start Back Screening Tool

Thinking about the **last 2 weeks**, check your response to the following questions:					
				Disagree<br>0	Agree<br>1
1.	My back pain has **spread down my leg(s)** at some time in the last 2 weeks			☐	☐
2.	I have had pain in the **shoulder** or **neck** at some time in the last 2 weeks			☐	☐
3.	I have only **walked short distances** because of my back pain			☐	☐
4.	In the last 2 weeks, I have **dressed more slowly** than usual because of my back pain			☐	☐
5.	It's not really safe for a person with a condition like mine to be physically active			☐	☐
6.	**Worrying thoughts** have been going through my mind a lot of the time			☐	☐
7.	I feel that **my back pain is terrible** and **it's never going to get any better**			☐	☐
8.	In general I have **not enjoyed** all the things I used to enjoy			☐	☐
9.	Overall, how **bothersome** has your back pain been in the **last 2 weeks**?				
	Not at all	Slightly	Moderately	Very much	Extremely
	☐<br>0	☐<br>0	☐<br>0	☐<br>1	☐<br>1
Total Score (all 9):			Sub Score (Q5–9):		

Scoring System:

Low Risk: Total Score = 3 or less
Medium Risk: Total Score = 4 or more + Sub Score Q5–9 = 3 or less
High Risk: Total Score = 4 or more + Sub Score Q5–9 = 4 or more

Validated prognostic tool used to screen primary care patients with acute low back pain to determine their risk of poor outcomes.
Adapted from Keele University: https://startback.hfac.keele.ac.uk/

© 2021 Stevans JM et al. *JAMA Network Open.*

eFigure 2. Algorithm to Classify Guideline Nonconcordant Care for Pharmacologic Therapies

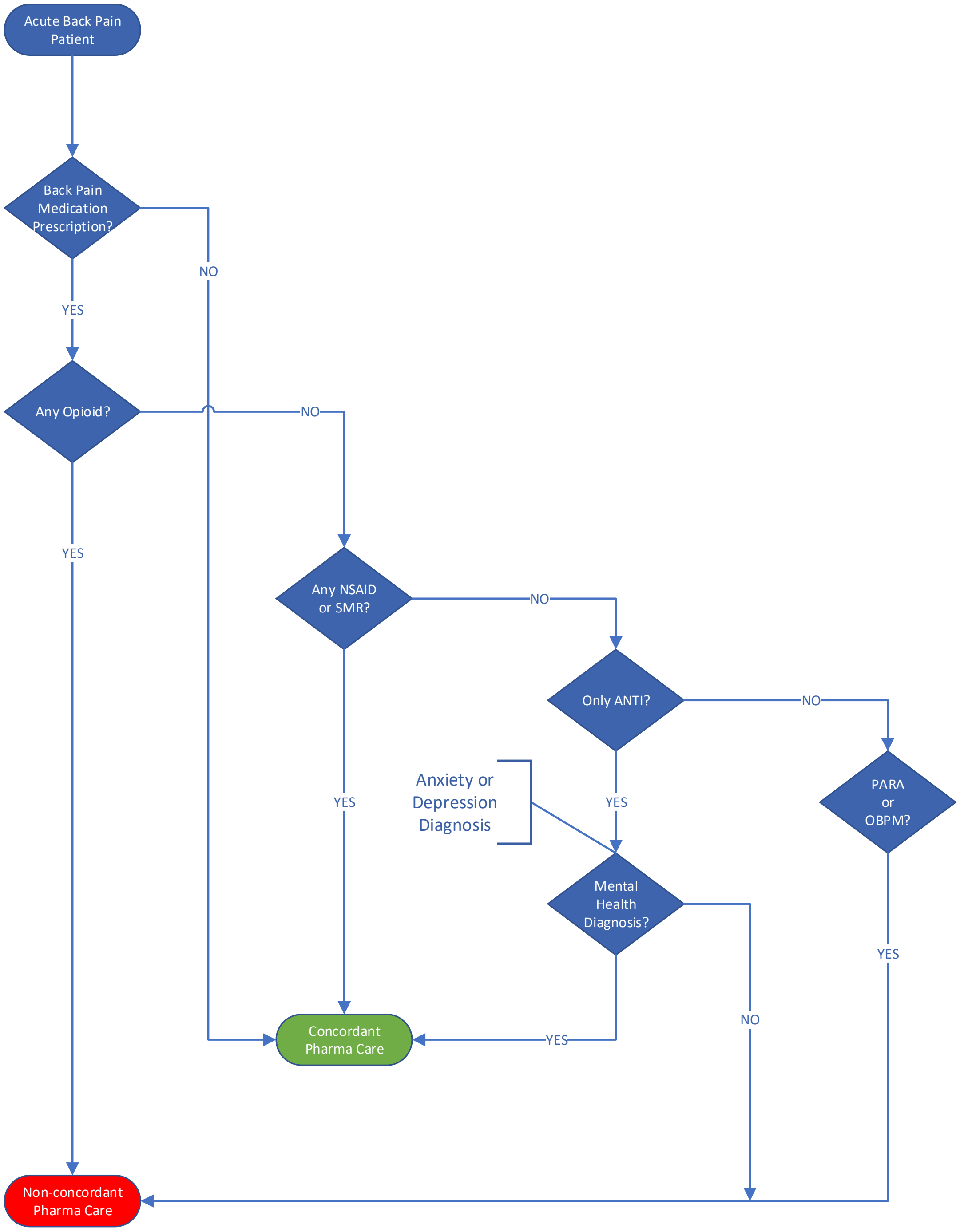

NSAID = Nonsteroidal Antiinflammatory Drugs
SMR = Skeletal Muscle Relaxants
ANTI = Antidepressants
OBPM = Systemic Corticosteroids or Benzodiazepines
PARA = Paracetamol/Acetaminophen

© 2021 Stevans JM et al. *JAMA Network Open.*
